# Correction: Changes in Innate and Permissive Immune Responses after HBV Transgenic Mouse Vaccination and Long-Term-siRNA Treatment

**DOI:** 10.1371/annotation/d0e70062-ac3b-47a6-ab1e-402ed08affa4

**Published:** 2013-06-18

**Authors:** Guang-Li Ren, Guang-Yu Huang, Hong Zheng, Ying Fang, Heng-Hao Ma, Man-Chun Xu, Hong-Bin Zhang, Wei-Yun Zhang, Ya-Gang Zhao, Da-Yong Sun, Wen-Kui Hu, Jian Liu

The word "Long" in the title is misspelled. The correct title is: Changes in Innate and Permissive Immune Responses after HBV Transgenic Mouse Vaccination and Long-Term-siRNA Treatment. The correct citation is: Ren G-L, Huang G-Y, Zheng H, Fang Y, Ma H-H, et al. (2013) Changes in Innate and Permissive Immune Responses after HBV Transgenic Mouse Vaccination and Long-Term-siRNA Treatment. PLoS ONE 8(3): e57525. doi:10.1371/journal.pone.0057525 

